# Immunohistochemical Experiments on Oral Mucoceles: A Narrative Review

**DOI:** 10.7759/cureus.91657

**Published:** 2025-09-05

**Authors:** Christina Charisi, Vasileios Zisis, Konstantinos Poulopoulos, Konstantinos Arapostathis, Nikolaos Dabarakis, Dimitrios Parlitsis, Athanasios Poulopoulos

**Affiliations:** 1 Oral Medicine/Pathology, Aristotle University of Thessaloniki, Thessaloniki, GRC; 2 Paediatric Dentistry, Aristotle University of Thessaloniki, Thessaloniki, GRC; 3 Oral Medicine/Pathology, European University Cyprus, Nicosia, CYP; 4 Dentoalveolar Surgery, Implantology and Oral Radiology, Aristotle University of Thessaloniki, Thessaloniki, GRC

**Keywords:** cyst, extravasation, immunohistochemistry, mucocele, mucous, retention, salivary

## Abstract

Oral mucoceles are among the most common benign salivary gland lesions, characterized by mucus extravasation or retention within the oral mucosa. This review aims to collect findings regarding the application of immunohistochemistry (IHC) in mucoceles and evaluate its efficiency. An electronic literature search was performed to identify all articles investigating IHC in oral mucoceles. The search was conducted using MEDLINE (National Library of Medicine)-PubMed, ScienceDirect-Elsevier, and the Cochrane Library without restrictions concerning the date of publication, using the following keywords: ((oral mucocele) OR (lip mucocele)) AND (immunohistochemistry). This was followed by a manual search, and references were screened to identify additional relevant articles.

The review showed no mucocele staining of HPV16 L1 and B19V VP1/VP2. MMP-1, MMP-2, MMP-9, α-SMA, mast cell tryptase, CD68, CD34, MCM3, p27, HBD-1, -2, and -3, D2-40, AGEs, IGF-1, IR, Ki-67, and CEA were present to some extent in the samples. Human DNA mismatch repair proteins hMSH2, hMLH1, and p53 protein were increased post-bone marrow transplantation. Type IV collagenases and plasminogen activators were present as well.

Accurate diagnosis and differentiation from other oral lesions relies on conventional histopathology, while IHC assists in atypical or recurrent cases. IHC employs epithelial, inflammatory, and mucin-specific markers to enhance diagnostic precision. There may be differences in the immunohistochemical profile between mucoceles in pediatric and adult populations, as well as between mucous retention cysts and mucous extravasation cysts. Novel biomarkers may shed light on mucocele pathogenesis and improve diagnostic accuracy.

## Introduction and background

Oral mucoceles exhibit common histopathological characteristics that reflect their pathogenesis and clinical presentation. Typically, these lesions are characterized by mucus extravasation, in which the lumen is filled with mucinous material and mucus-filled cells, often surrounded by granulation tissue, a feature that aids in differentiation from other cystic lesions of the oral cavity [1]. The granulation tissue forms as a reparative response to the extravasated mucin, and its prevalence and organization can vary, correlating with the chronicity and depth of the lesion [1,2]. Another diagnostic feature is the presence of salivary gland tissue and sialomucin within the lesion [1].

The epithelial lining may be absent, or in some cases replaced by hyperplastic parakeratinized stratified squamous epithelium or granulation tissue, due to remodeling processes that occur in response to repeated rupture and reaccumulation of mucin [1,3]. Additionally, long-standing mucoceles may manifest fibrotic changes on their surface as a result of chronic injury and repair [3]. Histological examination may differentiate between mucous extravasation and retention by the presence or absence of an epithelial lining around the cyst. In mucous retention cysts, the main causative factor is obstruction of the salivary gland duct, and a true epithelial lining is observed [4]. In contrast, in mucous extravasation cysts, the main causative factor is trauma, and thus a cyst-like space is created, filled with mucin and inflammatory infiltrate but without any epithelial lining, surrounded by connective and granulation tissue [4].

In chronic mucoceles, prolonged inflammation leads to the gradual loss of salivary gland architecture, impairing its function [5]. Over time, continuous irritation and inflammation may replace the normal oral mucosa with a fibrinopurulent membrane [5]. However, acute trauma may superimpose on chronic lesions, leading to an influx of inflammatory infiltrate within the chronic inflammatory microenvironment [5].

This review aims to collect findings regarding the application of immunohistochemistry (IHC) in mucoceles and evaluate its efficiency.

## Review

**Figure 1:**
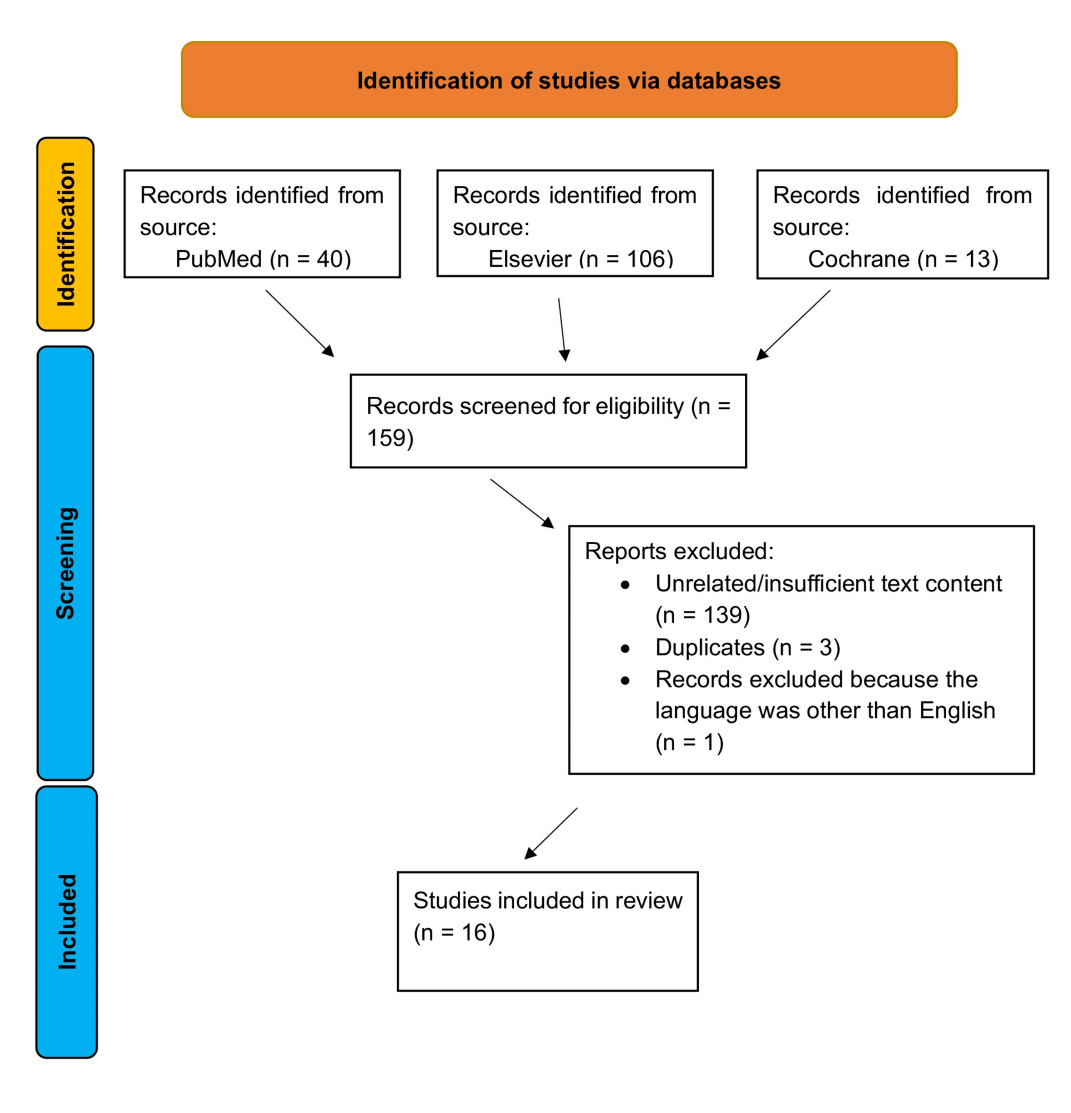
Flowchart of the screening process.

An electronic search of the literature was performed to identify all articles investigating IHC in oral mucoceles. The search was conducted using MEDLINE (National Library of Medicine)-PubMed, ScienceDirect-Elsevier, and the Cochrane Library without any restriction concerning the date of publication, using the following keywords: ((oral mucocele) OR (lip mucocele)) AND (immunohistochemistry). The search spanned from 1986 to 2025. A subsequent manual search identified additional relevant articles as per the aim of our review. Articles identified from the electronic and manual search were screened to eliminate those that failed to meet the respective inclusion criteria. The inclusion criteria referred to language (only articles written in English were analyzed) and type of study (only original research articles were analyzed). In total, 159 articles were identified through the keywords. Subsequently, by reading the titles and abstracts, and by excluding articles written in a language other than English as well as duplicates, 16 articles remained (Figure 1).

IHC markers may distinguish oral mucoceles from other histologically similar lesions. Markers for type IV collagenases [6] characterize the extracellular matrix. Markers such as CD138, Kappa, and CD20 exclude lymphoproliferative disorders and characterize the immune cell infiltrate [7]. LC3B staining enables the investigation of autophagy-related pathways and cellular turnover within the lesion [8]. Cytological examination constitutes another option since extracellular mucin may be collected in aspirate samples (mucinous cysts produce abundant mucin compared to non-mucinous lesions) [9]. The subsequent processing affects the observance of mucin as well; liquid-based cytology tends to diminish the visualization of mucin, risking possible misdiagnosis [9]. IHC agents such as alcian blue and mucicarmine confirm the presence of mucin, but in the case of GI contaminants, they may mimic mucin and disturb the diagnostic process [9]. Diagnostic errors are possible due to the overlap of cytologic features between mucoceles and other salivary lesions, such as atypical ductal hyperplasia, or benign and malignant neoplasms, which may complicate the differential diagnosis [10]. Additionally, sampling errors during scalpel biopsy, inadequate specimen preparation, or inadequate clinical-pathological correlation may complicate the diagnostic process as well [11]. Marker expression discrepancies are expected between pediatric and adult mucoceles [12,13]. Their distribution and clinical presentation may also vary depending on the age group [12]. This discrepancy results from differences in mucosal immune response, glandular development, and healing capacity [13]. The main causative factor also differs: trauma in children versus ductal obstruction in adults, affecting the molecular profile of mucoceles [13]. Understanding these interconnections is crucial, as it can inform age-specific diagnostic and therapeutic approaches, emphasizing the need for tailored interventions that consider both the clinical presentation and underlying marker expression profiles in pediatric and adult patients with mucoceles [12,13]. Age-related tissue responses influence the immunohistochemical profile. For instance, the accumulation of advanced glycation end products (AGEs) in the eye, particularly in Bruch’s membrane (a thin, five-layered structure separating the retina from the choroid), offers an in vitro model for mimicking aging and assessing the impact on cellular behavior and gene expression in retinal pigment epithelial cells [14]. Age-related substrate alterations are associated with mRNA changes, detected by microarray analyses and qRT-PCR, and affect the immunohistochemical profile [14]. Age-related cellular alterations include disrupted lysosomal enzyme activity and increased lipofuscin accumulation, both detectable by IHC [14]. Structural and histopathological features of mucoceles are better distinguished through immunohistochemical markers that identify granulation tissue and epithelial characteristics [15]. Innovative microscopy may also be applied. For example, multiphoton intravital microscopy provides higher spatial resolution and real-time visualization of immunohistochemical markers within living tissue, allowing mapping of protein distributions and cellular interactions in the tissue microenvironment [16].

The features of the included articles are summarized in Table 1.

**Table 1:** A summary of the studies included in the review, displaying the country where the study took place, the year of publication, the number of mucocele samples included in the experiment, and the conclusions derived from the experiment. IHC: Immunohistochemistry; HPV: Human Papillomavirus; B19V: Parvovirus B19; MMP: Matrix Metalloproteinase; MCM: Minichromosome Maintenance.

Study	Country	Year	Location of the study	Type of study	Cases of mucoceles	Conclusions
[17]	Ecuador	2024	University clinic	IHC	17	Absent HPV16 L1 staining
[18]	Iran	2023	University clinic	IHC	40	Absent B19V VP1/VP2 staining
[19]	Brazil	2015	University clinic	IHC	20	MMP-1, MMP-2, MMP-9, α-smooth muscle actin, and Ki-67 stained the samples to some extent
[20]	Brazil	2014	University clinic	IHC	32	Mast cell tryptase, CD68, MMP-1, MMP-9, and CD34 stained the samples to some extent. Mast cells and MMP-1 (p = 0.03); macrophages and MMP-1 (p = 0.01)
[21]	Brazil	2014	University clinic	IHC	11	MCM3, Ki-67, and p27 stained the samples to some extent
[22]	Japan	2008	University clinic	IHC	21	Human beta-defensin 1, 2, and 3 observed in the majority of the samples, stronger at the center than the periphery
[23]	Japan	2007	University clinic	IHC	23	Dilatation of lymphatics (observed through D2-40), caused by absorbing extravasated saliva, plays a role in the pathogenesis of mucoceles
[24]	Japan	2005	University clinic	IHC	15	Monocyte/macrophage lineage cells are a major source of endothelial cells in mucoceles through the production of tenascin, which mediates their differentiation
[25]	USA	2004	University clinic	IHC	15	Advanced glycation end product positive in all samples
[26]	Brazil	2004	University clinic	IHC	20	Human DNA mismatch repair proteins hMSH2, hMLH1, and p53 protein elevated post–bone marrow transplantation
[27]	USA	2003	University clinic	IHC	7	Stronger expression of the insulin receptor in Sjögren syndrome than in mucoceles, whereas the opposite was observed for insulin-like growth factor-1
[28]	USA	2000	University clinic	IHC	20	Human beta-defensin 1 protects salivary glands from retrograde infection; its expression is enhanced in inflammation, suggesting post-transcriptional regulatory mechanisms may be involved
[29]	Greece	2000	University clinic	IHC	20	Very weak staining of insulin-like growth factor-1 in mucoceles compared with Sjögren syndrome
[6]	Japan	1995	University clinic	IHC	4	Type IV collagenases and plasminogen activators detected, suggesting that proteolytic enzymes are involved in the pathogenesis of mucoceles
[30]	Japan	1986	University clinic	IHC	14	Mucoceles caused by ductal obstructions; keratin protein epithelial markers identified in ductal segments
[31]	Japan	1986	University clinic	IHC	20	Mucoceles accumulated carcinoembryonic antigen

Discussion

In 2024, Zambrano TB et al. concluded that HPV16 L1 expression in oral biopsies was relatively low, and specifically in oral mucocele samples the expression was absent [17]. The number of mucoceles included was relatively small; therefore, caution is necessary when interpreting the results in a clinical setting. In 2023, Abuei H et al. concluded that B19V VP1/VP2 staining was absent in oral mucoceles [18]. In this study, the number of samples was deemed satisfactory. In both of these studies, the virus was absent, meaning two things: (1) the virus is not implicated in the pathogenesis, and (2) in terms of routine biopsy, it is not possible to detect such viruses in clinically asymptomatic cases.

In 2015, Bianco BC et al. concluded that, to different extents, matrix metalloproteinase-1 (MMP-1), MMP-2, MMP-9, α-smooth muscle actin, and Ki-67 stained the 20 samples of oral mucoceles [19]. Such a number of samples may be considered borderline. When investigating relatively common oral disorders, at least 30 samples are required so that results may be supported with greater certainty. In 2014, two studies from Brazil showed that mast cell tryptase, CD68, MMP-1, MMP-9, CD34, MCM3, Ki-67, and p27 were present to some extent in oral mucocele samples [20,21]. However, only 11 samples were used for staining MCM3, Ki-67, and p27. Further studies with larger sample sizes are required to better elucidate the secretome of proinflammatory cytokines in such lesions.

In 2008, Frederic MK et al. showed that human beta-defensins 1, 2, and 3 are present in non-inflamed pseudocysts such as oral mucoceles, and in particular noted that staining was stronger at the center than at the periphery of the tissue specimen [22]. Human beta-defensins constitute antimicrobial peptides, are secreted by epithelial cells, and act as the first line of defense against bacteria, fungi, and viruses.

In 2007, Kundu S et al. documented dilatation of lymphatics (D2-40 staining), which was triggered by absorbing extravasated saliva, thereby playing a role in the emergence of mucoceles [23]. In 2005, Swelam W et al. revealed that endothelial cells forming new vessels were positive for CD31, CD34, vascular endothelial growth factor (VEGF), and von Willebrand factor (vWF) [24]. The vessels originating from neoangiogenesis were covered by tenascin and surrounded by foamy to epithelioid CD68-positive cells. Certain cells were also positive for CD34, VEGF, and one of its receptors, Flk-1. Such cells were aligned, which is typical of tubules [24]. Monocyte/macrophage cells were identified as progenitors of endothelial cells in mucous retention cysts due to a process called transdifferentiation, with tenascin guiding endothelial cell differentiation [24].

In 2004, two studies revealed additional perspectives on mucoceles. Katz J et al. observed AGE products present in all samples (with no difference between mucoceles and Sjögren syndrome at the immunohistochemical level, although the sample size was small) [25]. Gomez RS et al. concluded that human DNA mismatch repair proteins hMSH2, hMLH1, and p53 protein were elevated post-bone marrow transplantation in lip biopsy material [26]. Both studies suggest that salivary tissue is affected by systemic conditions and could potentially be used for individualized monitoring.

In 2003, Katz J et al. reported stronger expression of the insulin receptor in Sjögren syndrome than in mucoceles, whereas the opposite was observed for insulin-like growth factor-1 (though the sample size was small) [27]. This pattern suggests that mucoceles may serve as a baseline for comparison of immunohistochemical staining of growth factors in salivary disorders. In 2000, two studies by Sahasrabudhe KS et al. and Markopoulos AK et al. showed that human beta-defensin 1 protects salivary glands from retrograde infection, with its expression enhanced during inflammation, suggesting that post-transcriptional regulatory mechanisms may be involved in its expression [28-29]. Additionally, very weak staining of insulin-like growth factor-1 was noticed in mucoceles compared with Sjögren syndrome [29]. 

In 1995, Azuma M et al. detected type IV collagenases and plasminogen activators in a very small number of mucoceles, indicating that proteolytic enzymes are involved in their pathogenesis [6]. Finally, two studies in 1986 showed that mucoceles accumulated carcinoembryonic antigen and that ductal obstructions may be associated with specific keratin protein epithelial markers [30,31]. In such cases, the immunohistochemical profile of mucoceles and labial salivary gland tissue may be indicative of the general medical status of the patient. With regard to carcinoembryonic antigen, it is a protein that increases in certain cancers such as colorectal cancer, and minimally invasive excision of salivary tissue could be used to monitor its level.

Certain patterns emerge from the existing body of literature: the presence of other viruses may be examined in salivary mucocele tissue. Proinflammatory molecules may also be detected either due to local inflammation or due to the overall medical status of the patient. This may be analyzed in conjunction with other findings, such as AGE products, which are formed when sugars react non-enzymatically with proteins or lipids, contributing to aging and diseases such as diabetes and kidney failure [32]. Inflammation affects the cell infiltrate, meaning that mucoceles present an excellent histological microenvironment to assess the differentiation and distribution of cells in relatively healthy tissue. The same applies to the study of endothelial cells, vessels, and lymphatics. Ethics dictate that completely healthy tissue cannot be removed from patients for the sole purpose of research [33]; therefore, we need a condition that necessitates biopsy and, at the same time, remains as close to normal as possible so that the study of cells and the microenvironment is feasible.

The point of analyzing markers in mucoceles serves three purposes. First, it calibrates the expression of markers in non-malignant conditions, so that researchers may have a point of reference when analyzing markers in samples of salivary malignancies. Second, mucoceles are subdivided based on the depth of their localization, the presence or absence of epithelial lining, and the age group of patients. These parameters may correspond to a specific immunohistochemical profile, and this hypothesis necessitates further experimentation. Finally, natural cellular phenomena such as transdifferentiation may be studied in oral mucoceles due to the abundance of material originating from oral biopsies and the fact that the study takes place in a proinflammatory milieu rather than a malignant or potentially malignant one. This abundance of material is not possible when using normal oral mucosa, because it is unethical to remove completely healthy tissue solely for research purposes. Therefore, immunohistochemical research requires healthy-appearing mucosa, with a substrate as close to normal as possible, and mucoceles adhere to these standards. Last but not least, the immunohistochemical profile of mucoceles may reflect the overall medical situation of the patient, which may, to some extent, be attributed to the presence of an extended vascular and lymphatic network.

Future research

Based on the findings of this review, our forthcoming immunohistochemical experiments on oral mucoceles could focus on investigating novel biomarkers, such as different MMP molecules and markers related to blood vessels and lymphatics. Furthermore, age-related product markers could be investigated by dividing the mucoceles into groups based on patient age and comparing the presence of age-related products between, for example, adolescents and adults. The small sample size in most of the studies included in our review supports the need for investigating the aforementioned markers in larger sample sets so that the findings may be adequately supported.

Limitations

The limitations of this review include the exclusive analysis of mucoceles, excluding other salivary disorders, and the exclusive analysis of immunohistochemical experiments, excluding other experimental processes. The review was intentionally designed this way because it serves as the basis of our forthcoming experiments employing IHC in oral mucoceles.

## Conclusions

Lip trauma stimulates keratinocytes, which in turn secrete proinflammatory cytokines. In cases of salivary duct rupture, mucus spills into the tissue. This mucus provokes a response from macrophages, which secrete additional proinflammatory cytokines. The immunohistochemical profile of the secretome may depend on the presence of extravasation. Proinflammatory cytokines stimulate the production of matrix metalloproteinases by fibroblasts and promote neutrophil chemotaxis.

In this microenvironment, a variety of markers may be detected, some of which are associated with mucoceles, whereas others reflect the overall medical status of the patient. IHC can be employed both to characterize the mucocele and to provide valuable feedback regarding the patient’s overall health. Accurate diagnosis and differentiation from other oral and salivary lesions rely on conventional histopathology, while immunohistochemistry assists in atypical or recurrent cases by employing epithelial, inflammatory, and mucin-specific markers to enhance diagnostic precision.

There may be different immunohistochemical profiles between mucoceles in pediatric and adult populations, as well as between mucous retention cysts and mucous extravasation cysts. Novel biomarkers may shed light on mucocele pathogenesis and improve diagnostic accuracy.
